# Outcome of EGFR-mutated NSCLC patients with MET-driven resistance to EGFR tyrosine kinase inhibitors

**DOI:** 10.18632/oncotarget.21707

**Published:** 2017-10-09

**Authors:** Simon Baldacci, Julien Mazieres, Pascale Tomasini, Nicolas Girard, Florian Guisier, Clarisse Audigier-Valette, Isabelle Monnet, Marie Wislez, Maurice Pérol, Pascal Dô, Eric Dansin, Charlotte Leduc, Etienne Giroux Leprieur, Denis Moro-Sibilot, David Tulasne, Zoulika Kherrouche, Julien Labreuche, Alexis B. Cortot

**Affiliations:** ^1^ CHU Lille, Thoracic Oncology Department, Univ. Lille, Siric ONCOLille, Lille, France; ^2^ Toulouse University Hospital, Université Paul Sabatier, Toulouse, France; ^3^ Aix-Marseille University, Assistance Publique Hôpitaux de Marseille, Multidisciplinary Oncology & Therapeutic Innovations Department, Marseille, France; ^4^ Louis Pradel Hospital, Hospices Civils de Lyon, Lyon, France; ^5^ Rouen University Hospital, Thoracic oncology unit & Normandy University, IRIB, LITIS Lab, EA 4103 QuantIF team, Rouen, France; ^6^ Service de Pneumologie, Centre Hospitalier Sainte Musse, Toulon, France; ^7^ Centre Hospitalier Intercommunal de Créteil, Créteil, France; ^8^ APHP Hôpital Tenon, Paris, France; ^9^ Department of Medical Oncology, Centre Léon Bérard, Lyon, France; ^10^ Centre Régional de Lutte Contre le Cancer François Baclesse, Caen, France; ^11^ Centre Oscar Lambret, Lille, France; ^12^ CHU Strasbourg, Strasbourg, France; ^13^ APHP – Hôpital Ambroise Paré, Boulogne-Billancourt, France; ^14^ Unité d'Oncologie Thoracique, Service de Pneumologie, CHU Grenoble-Alpes, La Tronche, France; ^15^ Univ. Lille, CNRS, Institut Pasteur de Lille, UMR 8161, M3T, Mechanisms of Tumorigenesis and Targeted Therapies, Lille, France; ^16^ EA 2694 University of Lille, Lille, France

**Keywords:** non small cell lung cancer, EGFR, tyrosine kinase inhibitors, resistance, MET

## Abstract

**Background:**

Several mechanisms of acquired resistance to EGFR tyrosine kinase inhibitors (TKIs) in EGFR-mutated NSCLC have been described including the T790M mutation and *MET* amplification. Whereas T790M mutation confers prolonged survival and sensitivity to 3rd generation TKIs, data are lacking on clinical features and outcome of MET-driven resistant EGFR-mutated NSCLC patients.

**Methods:**

Patients with metastatic EGFR-mutated NSCLC displaying high MET overexpression or *MET* amplification, detected on a biopsy performed after progression on EGFR TKI, were identified in 15 centers. Clinical and molecular data were retrospectively collected.

**Results:**

Forty two patients were included. The median overall survival (OS), and the median post EGFR TKI progression overall survival (PPOS) were 36.2 months [95%CI 27.3-66.5] and 18.5 months [95%CI 10.6-27.4] respectively. Nineteen out of 36 tumors tested for MET FISH had *MET* amplification. A T790M mutation was found in 11/41 (26.8%) patients. T790M-positive patients had a better OS than T790M-negative patients (p=0.0224). Nineteen patients received a MET TKI. Objective response was reported in 1 out of 12 evaluable patients treated with a MET inhibitor as a single agent and in 1 of 2 patients treated with a combination of MET and EGFR TKIs.

**Conclusion:**

MET-driven resistance to EGFR TKI defines a specific pattern of resistance characterized by low objective response rate to MET inhibitors given alone and overlapping with T790M mutations. Further studies are warranted to define adequate therapeutic strategies for MET-driven resistance to EGFR TKI.

## INTRODUCTION

*EGFR* mutations are found in 10% of non small cell lung cancer (NSCLC) in Caucasians and 40% in Asians [1]. Treatment of advanced *EGFR*-mutated NSCLC patients relies on EGFR tyrosine kinase inhibitors (TKIs), which demonstrated superiority over chemotherapy as first-line therapy [2–5]. However, despite initial efficacy, all the patients will eventually develop resistance to EGFR TKIs resulting in tumor progression [6]. The most frequent mechanism of resistance is the T790M mutation, a second *EGFR* mutation that can be successfully targeted with third generation EGFR TKIs which have been specifically designed to overcome T790M-driven resistance [7, 8]

Besides T790M mutation, bypass activation of other tyrosine kinase receptors including MET or HER2 is the second most common mechanism of resistance to EGFR TKI. *MET* amplification leads to overexpression and constitutive activation of the receptor, thus activating the PI3K pathway and bypassing EGFR [9]. *MET* amplification has been detected in 5 to 22% of patients with an acquired resistance to EGFR TKI [9–14]. *MET* amplification is highly associated with high overexpression of MET in NSCLC (p<0.001) [15, 16]. Moreover, high MET overexpression with a 3+ immunoscore (IHC3+) by immunohistochemistry was recently found in 27% of *EGFR* mutated NSCLC with acquired resistance to EGFR TKI [17].

Little data is available about clinical characteristics and outcome of *EGFR*-mutated NSCLC patients with MET-driven resistance to EGFR TKIs. Moreover, optimal treatment of these patients is still unknown. Preclinical data and case reports suggest that *MET*-amplified *EGFR*-mutated NSCLC are addicted to both *MET* and *EGFR* and that combination of MET and EGFR TKIs is required to overcome this mechanism of resistance [9, 18, 19].

Whereas available data and specific treatments emerge for T790M-driven resistance in *EGFR*-mutated NSCLC patients, MET-driven resistance is still an unexplored field [20–22]. In the present study, we report clinical features, outcome and treatment in a series of *EGFR*-mutated NSCLC patients with MET-driven resistance to EGFR TKI.

## RESULTS

### Clinico-pathological and molecular characteristics

Forty six patients with metastatic NSCLC displaying both *EGFR* mutation and MET overexpression or *MET* gene amplification were retrospectively identified in 15 centers. Four patients were excluded : 3 had only a biopsy performed before EGFR TKI initiation, and 1 had no data available on the treatments received. Forty two patients were included. Re-biopsies of these patients had been performed from May 2011 to May 2016.

Patient clinical characteristics are summarized in Table 1. All 42 patients were diagnosed with metastatic lung adenocarcinoma. Median age was 65.1 years (range 30-82.7). The majority of patients were women (66.7%) and never smokers (70.7%). Most of the *EGFR* mutations detected on the initial biopsy were exon 19 deletions or exon 21 L858R point mutations.

**Table 1:** Patient characteristics

	Overall population	*MET* amplification	MET overexpression no *MET* amplification	p	T790M+	T790M-	p
	n = 42	n = 19	n = 17		n = 11	n = 30	
**Median age (years)**	65,1 (30-82,7)^*^	64,6 (30-74,6)^*^	65,6 (38,4-82,7)^*^	0,73	56,9 (45,2-70,3)^*^	67,4 (30-82,7)^*^	**0,038**
**Gender**				0,35			0,28
Men	14 (33,3%)	5 (26,3%)	7 (41,2%)	2 (18,2%)	12 (40%)		
Women	28 (66,7%)	14 (73,7%)	10 (58,8%)	9 (81,8%)	18 (60%)		
**Smoking status^†^**				0,45			1
Never smoker	29 (70,7%)	12 (63,2%)	12 (75%)		8 (72,7%)	20 (69%)	
Former and current smoker	12 (29,3%)	7 (36,8%)	4 (25%)	3 (27,3%)	9 (31%)		
**Stade IV**	42 (100%)	19 (100%)	17 (100%)		11 (100%)	30 (100%)	
**Adenocarcinoma**	42 (100%)	19 (100%)	17 (100%)		11 (100%)	30 (100%)	
**Initial EGFR mutation**				0,59			0,55
Exon 19 deletion or Exon 21 L858R mutation	39 (92,9%)	18 (94,7%)	15 (88,2%)		11 (100%)	27 (90%)	
Other^**^	3 (7,1%)	1 (5,3%)	2 (11,8%)		0 (0%)	3 (10%)	

^*^: range ; ^**^ : 1 exon 20 S768I mutation, 1 association of exon 21 L858R and K860I mutations, 1 association of exon 19 R761Y and exon 18 G719A mutations ; ^†^ : one missing data in total population ; TKI : tyrosine kinase inhibitor ; p : p value

The most frequent site of rebiopsy was the lung and re-biopsies were performed, in 90.5% of the cases, after the Response Evaluation Criteria in Solid Tumor (RECIST) progression on EGFR TKI, which was given as first or second line treatment (Supplementary Table 1). The median time between EGFR TKI initiation and re-biopsy was 15.6 months (range 2.1-61.3).

**Figure 1:**
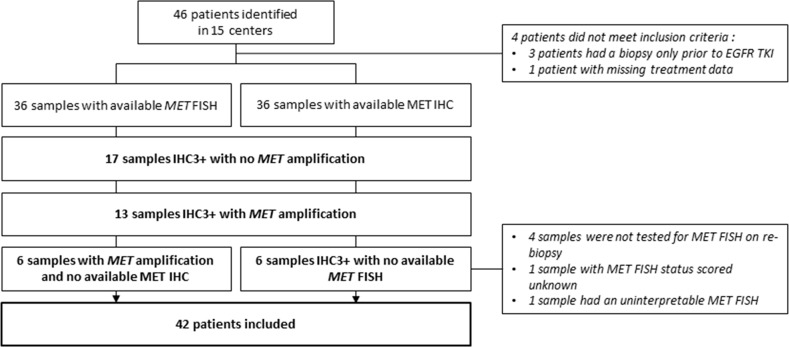
Flow chart of MET overexpression and MET amplification status on post EGFR TKI initiation sample IHC : Immunohistochemistry ; FISH : Fluorescence In Situ Hybridization ; TKI : tyrosine kinase inhibitor.

Among the 42 patients included in the study, 36 tumor re-biopsy samples were tested for *MET* FISH and 19 (52.8%) were found *MET* amplified (Figure 1). MET IHC was performed on the re-biopsy of 36 patients and all displayed a high level of MET expression (IHC3+). Six patients had a MET FISH but no MET IHC on their re-biopsy and conversely 6 other patients had MET IHC and no interpretable MET FISH. No difference was found regarding the *MET* amplified status between patients with an EGFR exon 19 deletion or an EGFR exon 21 mutation on the initial biopsy (Supplementary Table 2 ). Re-biopsies of 34 patients were tested for the BRAF mutations and none harbored a mutation of this oncogene. Otherwise no histological transformation in small cell lung cancer was reported among the 42 patients of the study.

### Clinical outcome and EGFR TKI treatment characteristics

Characteristics of initial EGFR TKI therapy are shown in Table 2. All patients received a first or second generation EGFR TKI. The overall response rate (ORR) was 82.1%, and the median progression free survival (PFS) was 11.1 months [95%CI 7.6-14.1]. In 73.2% of the cases, tumor progression involved a new lesion. The main site of new metastasis was the lung. 27 patients (66%) developed more than one progressive lesion at EGFR TKI resistance. The median post-progression overall survival (PPOS) and the median overall survival (OS) were respectively 18.5 months [95%CI 10.6-27.4] and 36.2 months [95%CI 27.3-66.5].

**Table 2:** EGFR TKI treatment characteristics

	Overall population	*MET* amplification	MET overexpression no *MET* amplification	p	T790M+	T790M-	p
	n = 42	n = 19	n = 17		n = 11	n = 30	
**First EGFR TKI received**				0,49			1
Erlotinib or Gefinitib	40 (95,2%)	17 (89,5%)	17 (100%)		11 (100%)	28 (93,3%)	
Afatinib	2 (4,76%)	2 (10,5%)	0 (0%)		0 (0%)	2 (6,7%)	
**Line of the first EGFR TKI therapy**				0,81			1
1	29 (69%)	13 (68,4%)	11 (64,7%)		8 (72,7%)	20 (66,7%)	
2	13 (31%)	6 (31,6%)	6 (35,3%)		3 (27,3%)	10 (33,3%)	
**Response to the EGFR TKI ^†^**				1			0,17
Objective response	32 (82,1%)	14 (77,8%)	12 (80%)		10 (100%)	22 (78,6%)	
Stable disease and Progression	7 (17,9%)	4 (22,2%)	3 (20%)		0 (0%)	6 (21,4%)	
**Median duration of TKI EGFR therapy (months)^‡^**	13,1 (1,4-52,5)^*^	13,8 (4,1-21,6)^*^	10,2 (1,4-45,4)^*^	0,61	14,0 (7,7-25)^*^	10,7 (1,4-52,5)^*^	0,51
**EGFR TKI progression involving a new metastasis ^‡^**	30 (73,2%)	14 (77,8%)	12 (70,6%)	0,71	8 (80%)	22 (73,3%)	1
**EGFR TKI progression involving more than one progressive lesion** ^‡^	27 (65.8%)	12 (63.2%)	11 (68.8%)	1	10 (90,9%)	16 (55.2%)	0.065

^*^: range ; TKI :tyrosine kinase inhibitor ; p : p value ; ^†^ : 3 missing data ; ^‡^ : one missing data

**Figure 2:**
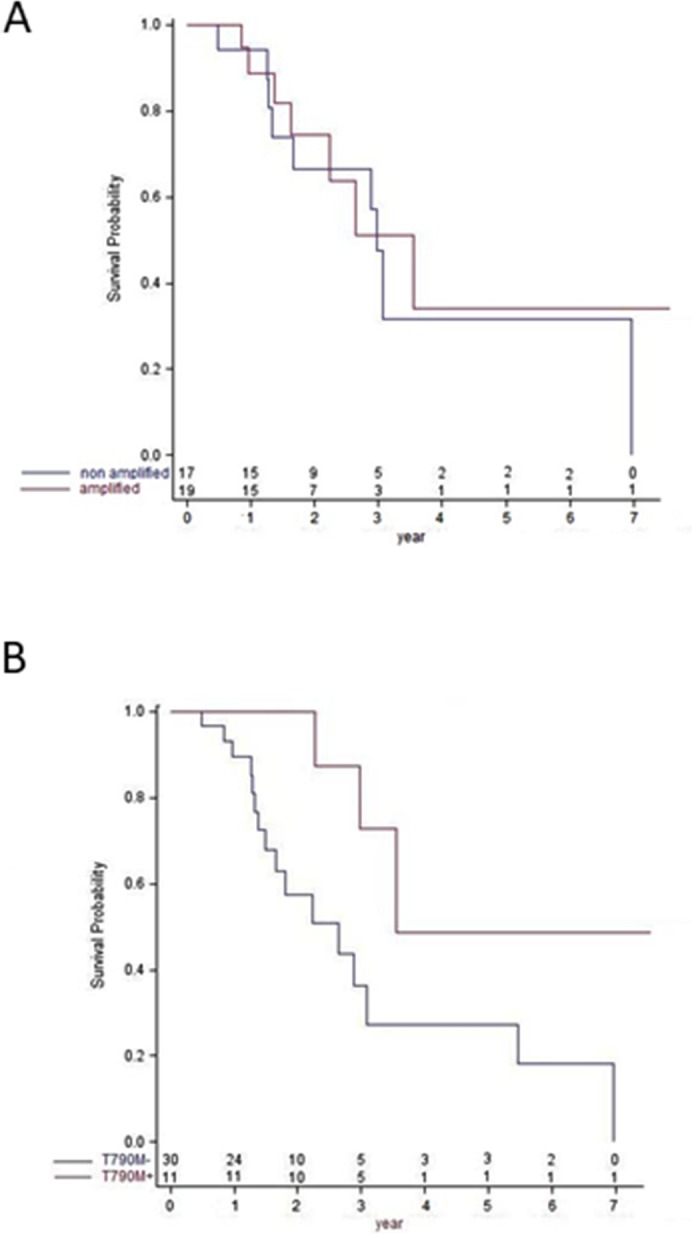
Overall survival according to T790M status and *MET* amplification status **(A)** Kaplan Meier estimates of overall survival in *MET* non amplified (blue) and *MET* amplified (red) patients. **(B)** Kaplan Meier estimates of overall survival in T790M-negative (blue) and T790M-positive (red) patients.

The characteristics of the EGFR TKI therapy were not significantly different according to the MET FISH status. No significant difference was found between patients with *MET* amplification and those with MET overexpression and no *MET* amplification in terms of OS (median OS : 42.8 vs. 36.2 months p = 0.69 ; Figure 2A), PPOS (median PPOS : 13.7 vs. 23.8 months p =0.77) and PFS on EGFR TKI (median PFS : 10.5 vs. 10.1 months p = 0.08). There were also no significant differences between the patients with EGFR exon 19 deletion on the initial biopsy and the patients with EGFR exon 21 mutation in terms of OS (median OS : 36.2 vs. 27.3 months p = 0.33), PPOS (median PPOS 18.5 vs. 12.6 months p = 0.91), and PFS on EGFR TKI (median PFS : 11.7 vs. 9.2 months p = 0.56). Of note only one patient with an EGFR exon 19 deletion received afatinib, and this drug was not used in patients with EGFR exon 21 mutation (Supplementary Table 2).

### Impact of the T790M mutational status

Eleven among the 41 patients (26.8%) tested for the T790M mutation were T790M positive. In 9 patients, the T790M mutation was detected in the tumor re-biopsy displaying MET overexpression or *MET* amplification. In the remaining 2 patients, the T790M mutation was detected in circulating free DNA in one case and in a tumor biopsy obtained before the re-biopsy displaying MET overexpression and *MET* amplification in another case. Three patients had both *MET* amplification and T790M mutation in the same sample. The T790M-positive patients were significantly younger than T790M-negative patients and the time between EGFR TKI initiation and re-biopsy was significantly longer in T790M positive patients (Table 1 and Supplementary Table 1).

T790M-positive patients had a better OS (median OS 43.1 vs. 32.2 months, p=0.0224) than T790M-negative patients (Figure 2B). There was also a trend to a better PPOS in T790M-positive patients compared to the T790M-negative patients although it did not reach statistical significance (median 23.8 vs. 11.0 months, p=0.075, Supplementary Table 3).

### Treatment with MET inhibitors and third generation EGFR TKI therapy

**Figure 3:**
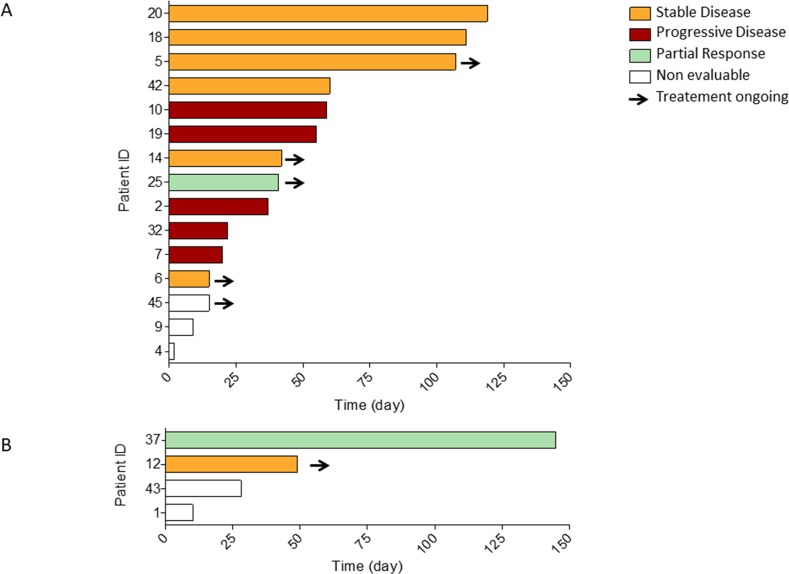
Duration of treatment with MET inhibitors **(A)** Patients treated with a MET inhibitor as a single agent; **(B)** Patients treated with a MET inhibitor in combination with an EGFR TKI. *ID* : identification number, Red: progressive disease as best response according to RECIST criteria ; Orange : stable disease as best response according to RECIST criteria ; Green : partial response as best response according to RECIST criteria, White : no tumor response evaluation available ; Arrow indicates that the MET inhibitor therapy is still ongoing.

Nineteen patients received a MET inhibitor, mostly crizotinib, including 13 *MET*-amplified patients (Table 3). The MET inhibitor was given as a monotherapy in 15 patients and in combination with an EGFR TKI in 4 patients. The MET inhibitor was used as 2^nd^ or 3^rd^ line in most of the cases. Objective response was reported in 1 out of 12 evaluable patients treated with a MET inhibitor as single agent and in 1 out of 2 evaluable patients treated with a combination of MET and EGFR inhibitors (Figure 3A and 3B). The MET inhibitor was stopped because of elevated liver enzymes in 2 patients and diarrhea in one patient. Two of these patients received the MET inhibitor in combination with an EGFR TKI. The median times between re-biopsy and MET inhibitor initiation and first EGFR TKI withdrawal and MET inhibitor initiation were respectively 2.3 months and 3.1 months.

**Table 3:** MET inhibitor tumor response

ID	*MET* amplification	IHC MET 3+	T790M	MET inhibitor	EGFR TKI in combination therapy	Line	RECIST Response	MET inhibitor status	Duration of MET inhibitor (days)
1	-	+	-	crizotinib	gefitinib	2	-	stopped (toxicity)	10
2	+	+	-	crizotinib	-	5	PD	stopped (PD)	37
4	+		-	crizotinib	-	4	-	stopped (patient's decision)	2
5	+		+	crizotinib	-	5	SD	ongoing	107
6	+	+	-	crizotinib	-	2	SD	ongoing	15
7	+	+	-	crizotinib	-	3	PD	stopped (PD)	20
9	+	+	-	crizotinib	-	2	-	stopped (toxicity)	9
10	+	+	-	crizotinib	-	3	PD	stopped (PD)	59
12	-	+	-	other	other	2	SD	ongoing	49
14	+		-	crizotinib	-	2	SD	ongoing	42
18	+		+	crizotinib	-	4	SD	stopped (PD)	111
19	+	+		crizotinib	-	4	PD	stopped (PD)	55
20	-	+	-	other	-	6	SD	stopped (PD)	119
25	+		-	crizotinib	-	2	PR	ongoing	41
32	-	+	-	other	-	3	PD	stopped (PD)	22
37	+	+	-	other	other	3	PR	stopped (PD)	145
42	-	+	-	crizotinib	-	6	SD	stopped (PD)	60
43		+	-	crizotinib	geftinib	4	-	stopped (toxicity)	28
45	+	+	+	crizotinib	-	4	-	ongoing	15

ID : identification number ; IHC : Immunochemistry ; PD : progressive disease ; SD : stable disease ; PR : partial response ; TKI : tyrosine kinase inhibitor ; RECIST : response evaluation criteria in solid tumor

Ten patients received a 3^rd^ generation EGFR TKI (Table 4) mostly osimertinib. Two partial responses were reported among the 5 T790M-positive evaluable patients who were treated following detection of MET overexpression or *MET* amplification. 3 patients were treated with 3^rd^ generation EGFR TKI before they received a MET inhibitor.

**Table 4:** Third generation EGFR TKI tumor response

ID	*MET* amplification	IHC MET 3+	T790M	Re-biopsy performed before 3^rd^ G EFGR TKI therapy	3^rd^ G EFGR TKI	Line	RECIST Response	3^rd^ G EFGR TKI status	Duration of 3^rd^ G EFGR TKI (days)
5	+		+	+	osimertinib	3	PD	stopped (PD)	48
16	-	+	+	+	other	2	PR	stopped (toxicity)	56
18	+		+	+	osimertinib	5	PD	stopped (PD)	80
20	-	+	-	+	osimertinib	5	PR	stopped (PD)	157
22		+	+	+	osimertinib	2	PR	ongoing	315
26	-	+	+	+	osimertinib	3	PD	stopped (PD)	84
27		+	+	+	osimertinib	4	-	ongoing	15
28	+	+	+	+	osimertinib	2	-	ongoing	18
45	+	+	+	-	osimertinib	3	SD	stopped (PD)	288
46	+	+	+	-	osimertinib	4	PR	ongoing	277

ID : identification number ; IHC : Immunochemistry ; TKI : tyrosine kinase inhibitor ; RECIST : response evaluation criteria in solid tumor ; 3^rd^ G EGFR TKI : Third Generation EGFRTKI

## DISCUSSION

Bypass activation of tyrosine kinase receptors is a well described mechanism of resistance to EGFR TKIs in *EGFR*-mutated NSCLC. MET-driven resistance has been reported in up to 22% of patients with acquired resistance to EGFR TKIs [9]. Still, although the biological basis of this mechanism of resistance has been extensively studied, there is very little data on the clinical characteristics and outcome of patients with MET-driven acquired resistance to EGFR TKIs. In this multicenter retrospective study, we report for the first time clinical features, response to MET inhibitors and outcome of 42 metastatic *EGFR*-mutated NSCLC patients with *MET* amplification or MET overexpression, as assessed on a post-progression re-biopsy. Because of the very low number of patients with MET-driven resistance to EGFR TKIs, we performed a multicentric retrospective study. Therefore, we could not perform a central analysis of RECIST responses, *MET* FISH, and MET IHC analysis. The absence of *MET* FISH and MET IHC analysis on the initial biopsy is also challenging. Indeed, we cannot exclude that some patients might have a MET overexpression or a *MET* amplification before EGFR TKI therapy. Most patients with EGFR exon 19 deletions in our study received a first generation EGFR TKI and were not treated with afatinib. There were no specific clinical characteristics of the patients included in our study but we observed a low rate of objective response to MET inhibitors when used as a monotherapy and a substantial rate of concomitant T790M mutations, which were still partially associated with efficacy of 3^rd^ generation EGFR TKIs and favorable prognosis, even in association with MET overexpression or *MET* amplification.

In our study, *MET* amplification was defined using the criteria provided by Schildhaus et al. to define high-level *MET* amplification, i.e. as an average *MET* gene copy number (GCN) per cell ≥ 6 or a ratio MET/CEP7 ≥ 2 or the presence of MET clusters [15]. Indeed these criteria were already widely used across our pathological centers to define positive *MET* FISH. However, many other definitions of *MET* amplification based on FISH have been proposed based on various thresholds for MET/CEP7 ratio or mean *MET* GNC [21, 23–27]. Unlike the mean *MET* GNC, the ratio MET/CEP7 is thought to discriminate real amplification from polysomia. Recent data showed that a MET/CEP7 ratio >5 was able to discriminate lung adenocarcinoma with no other driver mutations and was associated with high objective response rate to crizotinib [27, 28]. However, the right definition of a positive FISH that would allow identification of *MET* gene amplification remains to be determined. The ability to detect *MET* amplification through next generation sequencing may favor routine screening and harmonization of the definition of *MET* amplification.

In our study, *EGFR* mutated NSCLC patients with either *MET* amplification or MET overexpression (MET IHC3+) were included. In lung adenocarcinoma, *MET* amplification is significantly associated with IHC3+ MET overexpression [16]. In our study, all the patients with a *MET* amplification who also underwent a MET IHC were scored 3+. MET overexpression, regardless *MET* amplification status, has been found to induce addiction to the MET pathway, and was recently found in 27.1% of *EGFR* mutated NSCLC with acquired resistance to EGFR TKI [29] [17]. Moreover, an ongoing clinical trial evaluating the efficacy of combining MET and EGFR TKIs in *EGFR*-mutated NSCLC patients with MET-driven resistance to EGFR TKIs includes patients with both *MET* amplification or MET overexpression (NCT01610336). In patients without *MET* amplification, the cause of MET overexpression at the time of re-biopsy may involve other molecular alterations including MET exon 14 splicing sites mutations which have been reported to be associated with MET overexpression [30] and may be involved in resistance to EGFR TKI [31]. Because of the unavailability of most of the tumor samples, we could not test for other genomic alterations.

In our cohort, metastatic *EGFR* mutated NSCLC with a MET-driven resistance to EGFR TKI did not display specific clinical features compared to those observed in previous studies. Median PPOS (18.5 months) was also in the range of what has been reported in previous studies focusing on patients with acquired resistance to EGFR TKIs (14.3-20 months) [13, 32, 33]. Moreover, although *MET* GCN alterations and MET overexpression have been associated with poor prognosis in resected NSCLC [34, 35], we did not find poor outcome for the patients included in our study. These findings suggest that the prognostic impact of MET activation might depend on the stage of the disease and on the oncogenic environment. We observed only one objective response out of 12 patients treated with MET inhibitor monotherapy. In preclinical models, a double inhibition of EGFR and MET pathways was required to overcome MET driven resistance to EGFR TKI [9, 36]. Cases of *EGFR* mutated *MET* amplified NSCLC responding to combined MET and EGFR TKIs have been reported [18, 19]. This dual inhibition approach is currently evaluated in several phase I/II clinical trials in this setting using various EGFR (gefitinib, erlotinib, EGF816, osimertinib) and MET (capmatinib, volitinib, tepotinib) TKIs (NCT02468661, NCT01610336, NCT02335944, NCT02374645, NCT02143466, NCT01982955). Preliminary results from a phase II study, evaluating the combination of capmatinib and gefitinib in *EGFR* mutated NSCLC patients who progressed on EGFR TKI, reported an ORR of 19% in patients with MET IHC3+ or MET IHC≥2+ and *MET* GCN≥5 and raised to 30% in the subgroup of patients with *MET* GCN≥6 [37].

In our study, 11 patients were T790M positive. Three had both a *MET* amplification and a T790M mutation on the same sample. The coexistence of these mechanisms of resistance has been previously described [10]. Compared to the T790M negative, the T790M positive patients in our study had a better OS and a trend to a better PPOS. Several studies reported that, amongst patients with EGFR TKI acquired resistance, T790M-positive patients had a better PPOS than T790M-negative patients [13, 32, 38]. However, none of these studies evaluated the impact of concomitant *MET* amplification and overexpression. Of note, Gou *et* al. found that patients with a T790M mutation and MET overexpression had a worse PPOS than patients with only MET overexpression or the T790M mutation alone. This discrepancy with our results might be due to the small size of both cohorts, and variations in the characteristics of the study population (higher rate of never smokers and caucasians in our study). We cannot also exclude the influence of confounding factors such as the younger age and the use of 3^rd^ generation EGFR TKI in T790M positive patients. Indeed in our study, 9 T790M-positive patients received a 3^rd^ generation EGFR TKI and 3 partial responses were achieved. Very few data are available regarding the efficacy of 3^rd^ generation TKIs in the context of multiple resistance mechanisms. Our results suggest that 3^rd^ generation TKIs may retain activity against T790M-positive tumors in some patients, even in the presence of MET activation, which may be due to the spatial heterogeneity of resistance mechanisms rather than co-existence of 2 resistance mechanisms in the same tumor cells.

Finally, MET-driven resistance to EGFR TKI defines a specific pattern of resistance characterized by low objective response rate to MET TKIs given alone and overlapping with T790M mutations. Even when associated with MET dysregulation, the T790M mutation was still associated with relative efficacy of 3^rd^ generation EGFR TKIs and prolonged survival. Further studies are warranted to define adequate therapeutic strategies for MET-driven resistance to EGFR TKI.

## MATERIALS AND METHODS

### Patients

We constituted a multicenter retrospective observational cohort of patients identified among 15 French centers. Inclusion criteria were documented diagnosis of metastatic NSCLC, detection of an *EGFR* mutation on tumor sample at diagnosis, treatment with at least one EGFR TKI and detection of MET overexpression or *MET* amplification after the time of clinical or radiological progression on EGFR TKI. Post-progression re-biopsy and MET status assessment were routinely performed in participating centers during the study period. Clinical and pathological data were retrospectively collected in each center for all included patients. Best overall response, defined as the best response from the start of treatment until disease progression, was assessed by investigators from available follow-up exams in each center using Response Evaluation Criteria in Solid Tumor (RECIST) v1.1. Crizotinib, gefinitib, and osimertinib were respectively prescribed with the following doses and schedules : 250mg bid, 250mg daily, 80mg daily. The study was approved by a national ethic committee (CEPRO 2016-001).

### Histological and molecular analyses

Histological and molecular analyses of formalin-fixed paraffin-embedded (FFPE) samples were prospectively assessed by local pathologists in accredited and quality controlled laboratories, as part of the routine procedure. MET immunochemistry and *MET* FISH analyses were realized according to locally certified and nationally approved procedures. MET overexpression was defined as a 3+ MET Immunoscore (≥ 50% of tumor cells showing high-intensity staining) on MET immunochemistry using MET monoclonal antibody (clone SP44 Ventana). *MET* amplification was defined by Fluorescence In Situ Hybridization (FISH) as a mean *MET* GNC per cell ≥ 6, or a Ratio MET/CEP7 ≥ 2, or the presence of MET clusters [15]. Patients were considered “T790M positive” if an *EGFR* T790M mutation was detected in post-progression circulating tumor DNA, on the re-biopsy on which MET overexpression or *MET* amplification was found, or on a tumor sample collected before the re-biopsy.

### Statistical analyses

Categorical variables are expressed as frequencies and percentages. Quantitative variables are expressed as medians (range). Normality of distribution was assessed graphically and by using the Shapiro–Wilk test. Bivariate analyses were realized to assess the sub-group comparability (*MET* amplified *vs*. *MET* non-amplified and T790M+ *vs*. T790M-). Chi-Squared tests or Fisher's exact test (when expected cell frequency <5) were used to study the association between categorical variables and different groups. A Mann-Withney U test was used to compare age, time between EGFR TKI initiation and rebiopsy, and duration of TKI EGFR therapy between groups. We estimated and compared overall survival (OS), post progression overall survival (PPOS) and progression free survival (PFS) between the study groups (*MET* amplified *vs*. *MET* non amplified and T790M+ *vs*. T790M-) using the Kaplan-Meier Method and log-rank test. OS was measured from the date of metastatic NSCLC diagnosis to the date of death from any cause or last follow-up. The PPOS was measured as the time from EGFR TKI RECIST progression to death from any cause. The PFS was defined as the time from treatment start to disease progression or death from any cause. Objective response rate (ORR) was defined as the percentage of patients with partial or complete response to the indicated treatment. Statistical testing was conducted at the 2-tailed α level of 0.05. Data were analyzed with SAS software version 9.3 (SAS Institute, Cary, NC).

## SUPPLEMENTARY TABLES



## Abbreviations

NSCLC: Non small cell lung cancer
TKI: Tyrosine kinase inhibitor
OS: overall survival
PPOS: post progression overall survival
PFS: progression free survival
ORR: objective response rate
RECIST: response evaluation criteria in solid tumor
GNC: gene copy number
IHC: immunohistochemistry
FFPE: formalin-fixed paraffin-embedded
FISH: Fluorescence In Situ Hybridization
