# Supplementary material for: Outcome of EGFR-mutated NSCLC patients with MET-driven resistance to EGFR tyrosine kinase inhibitors
Source: Oncotarget. 2017 Oct 9;8(62):105103–14. doi: 10.18632/oncotarget.21707 (PMC5739624; doi:10.18632/oncotarget.21707)
Supplement: Supplementary file 1 [file oncotarget-08-105103-s001.pdf]

# Outcome of EGFR-mutated NSCLC patients with MET-driven resistance to EGFR tyrosine kinase inhibitors

## SUPPLEMENTARY MATERIALS

**Supplementary Table 1: Re-biopsy characteristics. * : range ; † : one missing data in total population ; TKI : tyrosine kinase inhibitor ; p : p value**

| | Overall population | *MET* amplification | MET overexpression no *MET* amplification | p | T790M+ | T790M- | p |
|---|---|---|---|---|---|---|---|
| | **n = 42** | **n = 19** | **n = 17** | | **n = 11** | **n = 30** | |
| **Re-biopsy obtained after EGFR TKI RECIST progression** | 38 (90,5%) | 15 (78,9%) | 17 (100%) | 0,11 | 10 (90,9%) | 27 (90%) | 1 |
| **Number of lines ongoing or over at the time of re-biopsy** | | | | 1 | | | 1 |
| ≤2 | 37 (88,1%) | 17 (89,5%) | 15 (88,2%) | | 10 (90,9%) | 26 (86,7%) | |
| ≥3 | 5 (11,9%) | 2 (10,5%) | 2 (11,8%) | | 1 (9,1%) | 4 (13,3%) | |
| **Median time between re-biopsy and EGFR TKI initiation (months)** | 15,6 (2,1-61,3)* | 14,1 (3,6-31,8)* | 20,7 (2,1-31,3)* | 0,39 | 22,1 (9,1-36)* | 12,1 (2,1-61,3)* | **0,050** |
| **Re-biopsy obtained while TKI EGFR is still ongoing†** | 28 (68,3%) | 15 (79,0%) | 9 (56,3%) | 0,15 | 5 (50%) | 23 (76,7%) | 0,13 |
| **Re-biopsy site** | | | | | | | |
| Lung | 23(54,8%) | 12(63,2%) | 8(47,1%) | | 6(54,5%) | 16(53,3%) | |
| Pleura | 3(7,1%) | 2(10,5%) | 1(5,9%) | | 0(0%) | 3(10%) | |
| Lymph node metastasis | 4(9,5%) | 1(5,3%) | 1(5,9%) | | 1(9,1%) | 3(10%) | |
| Brain | 3(7,1%) | 0(0%) | 3(17,6%) | | 1(9,1%) | 2(6,7%) | |
| Liver | 4(9,5%) | 2(10,5%) | 1(5,9%) | | 2(18,2%) | 2(6,7%) | |
| Skin | 1(2,4%) | 1(5,3%) | 0(0%) | | 0(0%) | 1(3,3%) | |
| Adrenal glands | 2(4,8%) | 0(0%) | 2(11,8%) | | 0(0%) | 2(6,7%) | |
| Bone | 1(2,4%) | 0(0%) | 1(5,9%) | | 1(9,1%) | 0(0%) | |
| Muscle | 1(2,4%) | 1(5,3%) | 0(0%) | | 0(0%) | 1(3,3%) | |

**Supplementary Table 2: First EGFR TKI therapy and MET alterations of patients with an EGFR exon 19 deletion or an EGFR exon 21 mutation on the initial biopsy.** [*]**: % calculated with the number of patients with a MET FISH ;** [**] **: % calculated with the number of patients with a MET IHC ; TKI : tyrosine kinase inhibitor ; IHC : Immunochemistry ; FISH : Fluorescence In Situ Hybridization ; p : p value**

| | Patients with EGFR exon 19 deletion | Patients with EGFR exon 21 L858R mutation | p |
|---|---|---|---|
| | N = 23 | N =16 | |
| **First EGFR TKI** | | | 1 |
| gefintib or erlotinib | 22 (95,7%) | 16 (100%) | |
| afatinib | 1 (4,3%) | 0 (0%) | |
| **MET FISH on re-biopsy** | 21 (91,3%) | 12 (75%) | 0,21 |
| ***MET* amplified on re-biopsy** | 12 (57,1%)[*] | 6 (50%)[*] | 0,73 |
| **MET IHC on re-biopsy** | 20 (87%) | 13 (81,25%) | 0,67 |
| **MET IHC 3+ on re-biopsy** | 20 (100%)[**] | 13 (100%)[**] | 1 |

**Supplementary Table 3: Molecular alterations and prognosis of T790M positive patients.** [*] **lost of follow up immediately after EGFR TKI progression.** [*] **patient 45 : the T790M mutation was not detected on the re-biopsy displaying MET amplification. However, very few tumor cells were found in this sample. The T790M mutation was detected in a tumor biopsy obtained before the re-biopsy displaying MET amplification in this patient.** [†] **patient 46 : the research of T790M mutation was not performed on the re-biopsy displaying MET amplification. The T790M mutation was detected in circulating free DNA in this patient. OS : overall survival. PPOS : post progression overall survival. OS and PPOS are expressed in months**

| ID | *MET* amplification | Primary EGFR mutation | OS | PPOS |
|---|---|---|---|---|
| 5 | + | Exon 19 deletion | 121,3 | 8,7 |
| 16 | - | Exon 21 L858R mutation | 26,7 | 16,0 |
| 18 | + | Exon 21 L858R mutation | 43,1 | 36,9 |
| 22 | | Exon 19 deletion | 36,6 | 14,6 |
| 26 | - | Exon 19 deletion | 36,2 | 23,8 |
| 27 | | Exon 21 L858R mutation | 25,0 | 17,0 |
| 28 | + | Exon 21 L858R mutation | 21,8 | 1,6 |
| 34[*] | - | Exon 19 deletion | 37,1 | 0 |
| 35 | | Exon 19 deletion | 27,7 | 13,7 |
| 45[**] | + | Exon 19 deletion | 33,8 | 18,6 |
| 46[‡] | + | Exon 19 deletion | 44,3 | 12,9 |
